# Barriers to uptake among high-risk individuals declining participation in lung cancer screening: a mixed methods analysis of the UK Lung Cancer Screening (UKLS) trial

**DOI:** 10.1136/bmjopen-2015-008254

**Published:** 2015-07-14

**Authors:** Noor Ali, Kate J Lifford, Ben Carter, Fiona McRonald, Ghasem Yadegarfar, David R Baldwin, David Weller, David M Hansell, Stephen W Duffy, John K Field, Kate Brain

**Affiliations:** 1 Cochrane Institute of Primary Care and Public Health, Cardiff University School of Medicine, Cardiff, UK; 2 Department of Molecular and Clinical Cancer Medicine, Institute of Translational Medicine, The University of Liverpool Cancer Research Centre, Liverpool, UK; 3 Respiratory Medicine Unit, David Evans Centre, Nottingham University Hospitals, Nottingham, UK; 4 Centre for Population Health Sciences, Medical School, Edinburgh, UK; 5 Department of Radiology, Royal Brompton Hospital, London UK; 6 Wolfson Institute of Preventive Medicine, Barts and the London School of Medicine and Dentistry, London, UK

**Keywords:** Lung cancer, Screening, Barriers, High-risk, UKLS

## Abstract

**Objective:**

The current study aimed to identify the barriers to participation among high-risk individuals in the UK Lung Cancer Screening (UKLS) pilot trial.

**Setting:**

The UKLS pilot trial is a randomised controlled trial of low-dose CT (LDCT) screening that has recruited high-risk people using a population approach in the Cambridge and Liverpool areas.

**Participants:**

High-risk individuals aged 50–75 years were invited to participate in UKLS. Individuals were excluded if a LDCT scan was performed within the last year, if they were unable to provide consent, or if LDCT screening was unable to be carried out due to coexisting comorbidities.

**Outcome measures:**

Statistical associations between individual characteristics and UKLS uptake were examined using multivariable regression modelling. In those who completed a non-participation questionnaire (NPQ), thematic analysis of free-text data was undertaken to identify reasons for not taking part, with subsequent exploratory linkage of key themes to risk factors for non-uptake.

**Results:**

Comparative data were available from 4061 high-risk individuals who consented to participate in the trial and 2756 who declined participation. Of those declining participation, 748 (27.1%) completed a NPQ. Factors associated with non-uptake included: female gender (OR=0.64, p<0.001), older age (OR=0.73, p<0.001), current smoking (OR=0.70, p<0.001), lower socioeconomic group (OR=0.56, p<0.001) and higher affective risk perception (OR=0.52, p<0.001). Among non-participants who provided a reason, two main themes emerged reflecting practical and emotional barriers. Smokers were more likely to report emotional barriers to participation.

**Conclusions:**

A profile of risk factors for non-participation in lung screening has emerged, with underlying reasons largely relating to practical and emotional barriers. Strategies for engaging high-risk, hard-to-reach groups are critical for the equitable uptake of a potential future lung cancer screening programme.

**Trial registration number:**

The UKLS trial was registered with the International Standard Randomised Controlled Trial Register under the reference 78513845.

**Strengths and limitations of this study**

- To the best of our knowledge, this study is the first to use a mixed methods approach to examine the barriers to participation among high-risk individuals in a lung cancer screening trial.
- The study highlighted important subgroups with low uptake of lung cancer screening and in whom lung cancer risk is known to be higher.
- Increasing uptake among these high-risk groups is key to implementing an equitable lung cancer screening programme.
- Methodological issues associated with response bias are acknowledged, whereby there was an under-representation of younger individuals, individuals from lower socioeconomic groups, and smokers in those completing the non-participation questionnaire.

## Introduction

Lung cancer is the leading cause of cancer death in the UK, with approximately 35 000 deaths a year.^1^ The overall 5-year survival rate is less than 10%, largely due to most patients presenting with late-stage disease when treatment has little effect on survival.^1^ Lung screening is not yet available as a routine screening programme in the UK, but is being evaluated in a pilot trial. The UK Lung Cancer Screening (UKLS) pilot trial compares a single low-dose CT (LDCT) scan with usual care.^2^ LDCT was introduced in the late 1990s and offers a major advance in imaging technology.^3^ It is more sensitive than chest X-ray and has enabled detection of small, asymptomatic lung tumours.^4^ ^5^ The US-based National Lung Cancer Screening Trial (NLST) showed that LDCT screening resulted in a 20% reduction in lung cancer-related mortality when compared with chest radiography.^6^ Results from the on-going Dutch-Belgian lung cancer screening trial (NELSON), investigating whether LDCT screening reduces mortality compared with no screening at 10 years follow-up, are yet to be published.

Compared with routine screening for other types of cancers, lung screening in the UKLS trial was only available to high-risk individuals as part of a two-stage population risk screening strategy. It has previously been shown that individuals from lower socioeconomic groups, smokers and younger individuals were less likely to take part at the first stage of recruitment to the UKLS trial.^7^ However, the factors affecting uptake of these initial recruits who were subsequently identified as being at high risk of developing lung cancer have not been examined.

Inequalities in participation present a significant challenge to the successful implementation of cancer screening programmes. Reduced uptake of participants for cancer screening has been consistently found among deprived populations and ethnic minority groups,^8^ ^9^ and previous studies have highlighted some of the barriers and facilitators to lung screening.^10–12^ Van den Bergh *et al*^10^ found that participants in the NELSON had a more positive attitude towards the benefits of lung cancer screening, as well as a higher affective risk perception, when compared with non-participants. Silvestri *et al*^11^ found that smokers were less likely to participate in screening than non-smokers, due to lower perceived effectiveness of lung cancer early detection strategies. A qualitative study described four typological behaviours among those declining participation in a lung cancer screening programme: individuals who felt they were ‘too old to benefit’, ‘avoiders’ who preferred not to know their lung cancer status, ‘worriers’ who felt that participation would increase their anxieties, and ‘fatalists’ who believed that if they were to develop lung cancer, this would occur regardless of being screened or not.^12^

The influence of affective risk perception on screening uptake is particularly relevant in the context of a targeted screening strategy to identify those at high risk of developing lung cancer. Affective risk perception refers to an individual's degree of concern or worry associated with personal risk, rather than a quantitative estimate of their risk.^13^ There is much debate about whether higher affective risk perception motivates or deters individuals from cancer screening. Studies including the NELSON have shown that individuals with a higher affective risk of lung cancer are more interested in taking part in screening.^14^ ^15^ However, a body of evidence regarding uptake of other forms of cancer screening suggests that a moderate level of affective risk perception optimises screening uptake, with too little creating a lack of motivation and too much leading to avoidance of screening.^16^ ^17^

The aim of the current study was to use a mixed methods approach to identify the barriers to uptake among high-risk individuals invited to participate in UKLS. We aimed to answer three questions: (1) What are the demographic and psychological characteristics of individuals declining participation at the second stage of the UKLS trial? (2) Among those declining and stating their reason, what are the reported barriers to participation? (3) Are there any associations between individual characteristics and self-reported barriers to participation? It was hypothesised that declining to take part in UKLS would be associated with lower socioeconomic group, smoking, and higher affective risk perception. Identifying barriers to participation among high-risk individuals in the UKLS trial will inform the implementation of a potential national lung cancer screening programme.

## Methods

### Procedures

UKLS is a multicentre randomised controlled pilot trial to compare the intervention of LDCT screening versus usual care for the early detection of lung cancer in high-risk individuals.^2^ Individuals from six primary care trusts (PCTs) in Liverpool and Cambridge were approached with an invitation letter and a participation questionnaire. The invitation packs were posted by the data management company using the respective PCT-headed notepaper. The Liverpool Lung Project (LLP) risk algorithm was used to identify individuals with >5% risk of developing lung cancer over 5 years.^18^ This model incorporates age, sex, family history of lung cancer, smoking duration, personal history of other cancers and non-malignant respiratory disease, and occupational exposure to asbestos.

High-risk individuals who consented to take part in the screening trial were referred to as ‘positive uptake’, while those who declined participation immediately following risk assessment were referred to as ‘non-uptake’. Positive uptake individuals were invited to a recruitment centre (at either Liverpool Heart and Chest Hospital or Papworth Hospital), where they were given further information about the trial, provided written informed consent, and completed a touchscreen questionnaire. Non-uptake individuals were asked to complete a paper-based optional non-participation questionnaire (NPQ) and return it using a freepost envelope attached. The NPQ contained six closed response items and one free-text item (see online supplementary appendix S1).

### Participants

High-risk individuals aged 50–75 years residing in six PCTs in the Cambridge and Liverpool areas, and able to provide written informed consent were included in the study. Individuals were excluded from the UKLS trial if a LDCT scan of the chest had been performed within the previous year of invitation or they were unable to provide consent. Individuals with comorbidities that contraindicated either screening or treatment if lung cancer was detected were also excluded, as were those unable to lie flat or weighing greater than 200 kg.

### Measures

#### Age and gender

Age and gender were provided by PCTs via the data management company RADAR. Age referred to age at time of risk calculation and was analysed using three categories: ≤65 years (younger), 66–70 years (recently retired) and ≥71 years (older).

#### Socioeconomic group

Participants’ postcodes were used by the data management company to provide Index of Multiple Deprivation (IMD) ranks. IMD ranks were analysed using standard quintiles based on England-wide population data—quintile 1: 1–6496; quintile 2: 6497–12 993; quintile 3: 12 994–19 489; quintile 4: 19 490–25 986 and quintile 5: 25 987–32 482.^19^ Quintile 1 reflects those most deprived (lowest socioeconomic group) and quintile 5 those least deprived (highest socioeconomic group).

#### Smoking status

Smoking status data were collected at the first stage of the UKLS trial, and analysed using three categories: ‘*current smoker*’, ‘*ex-smoker*’, and ‘*never-smoker*’. Very few high-risk participants had never smoked, hence this category was excluded during analyses.

#### Affective risk perception

Affective risk perception was measured using 1 item taken from the revised 6-item Cancer Worry Scale^20^ ^21^ and refers to the degree of concern associated with personal risk of lung cancer. Data were collected from the NPQ for NPQ completers, and from the touchscreen questionnaire at the recruitment centre for positive uptake individuals. Data were unavailable for NPQ non-completers. Participants were asked to rate how concerned they were about the possibility of getting lung cancer someday. Response options included ‘*not at all’*, ‘*somewhat’*, ‘*moderately’* and ‘*very’ concerned*. Three categories of affective risk perception were created: none (‘*not at all*’ concerned), lower (‘*somewhat*’ concerned), and higher (‘*moderately*’ or ‘*very*’ concerned).

### Reason for non-participation

Qualitative data regarding reason(s) for non-participation were gathered using an optional free-text question within the NPQ (“If you would like to tell us your reason for not taking part in the UKLS trial, please write it here”).

### Analyses

Associations between individual characteristics and screening uptake were analysed using univariable logistic regression. To determine the effect of missing data on results, sensitivity analyses of augmented data sets were undertaken. For each variable with missing data >5%, this involved coding the missing data as each potential response, and determining whether there was a difference in outcome between each augmented data set. If for each augmented data set there was no statistically significant difference in the outcome, missing data were determined as having no statistically significant effect on the pattern of results and were, therefore, excluded from analyses.

Demographic and psychological characteristics found to have statistically significant associations in univariable analyses (p<0.05) were included in a multivariable logistic regression to identify the key risk factors for non-uptake. Demographic differences between NPQ completers and NPQ non-completers were compared using univariable associations. Statistical analyses were conducted using STATA V.12.

In individuals who provided a reason for non-uptake, free-text data were analysed to identify underlying themes.^22^ A random sample of 25% of the data was independently coded by another researcher (KJL), and discrepancies were resolved by discussion to reach consensus. NVivo V.10 was used to manage the data. Finally, exploratory regression analyses were undertaken to assess associations between key risk factors and key themes (reported by >5% of non-uptake individuals) in high-risk individuals who declined screening.

## Results

### Trial participation

**Figure 1:**
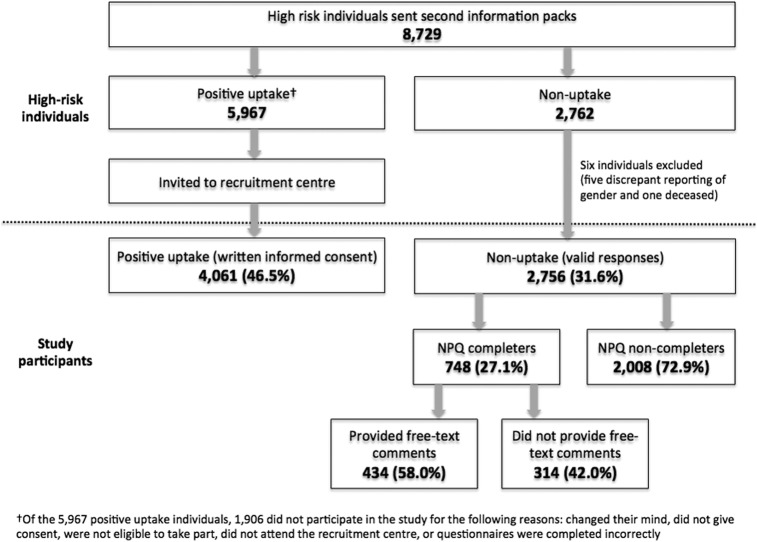
Consort diagram showing response rate and recruitment of high-risk individuals in the UKLS trial (NPQ, non-participation questionnaire; UKLS, UK Lung Cancer Screening).

Figure 1 shows the response rate and recruitment of high-risk individuals in the UKLS trial. Of the 2762 non-uptake individuals, five individuals were excluded due to reported gender discrepancies and one individual was reported as deceased. Therefore, of the 8729 high-risk individuals, the current study included 4061 (46.5%) individuals who consented to participate in the UKLS trial and 2756 (31.6%) who declined participation. Among those declining, 748 (27.1%) individuals completed the NPQ and of these 434 (58.0%) provided comments in the optional free-text field. Sensitivity analyses revealed that missing data had no statistically significant effect on the pattern of results.

### Factors influencing uptake among high-risk individuals

Age, gender, smoking status, and socioeconomic group were statistically significantly associated with lung cancer screening uptake (see table 1). Women were less likely to take part in screening compared with men (OR=0.64; p<0.001), and current smokers were less likely to take part than ex-smokers (OR=0.70, p<0.001). Older individuals were less likely to participate in screening compared with younger individuals aged ≤65 years (OR=0.73, p<0.001) and those recently retired (OR=0.76, p<0.001), but the difference in uptake between younger individuals and those recently retired was not statistically significant. Individuals in the highest socioeconomic group (quintile 5) were most likely to participate in screening. Individuals in the lowest quintile were almost twice as likely to decline screening compared with those in the highest quintile (OR=0.56, p<0.001).

**Table 1:** Univariable and multivariable analyses of factors influencing lung-screening uptake in high-risk individuals

	Non-uptake (n=2756) n (%)	Positive uptake (n=4061) n (%)	Univariable OR (95% CI)	p Value	Multivariable OR (95% CI)†	p Value
Gender						
Male	1770 (64.2)	3041 (74.9)	1.00 (REF)			
Female	986 (35.8)	1020 (25.1)	0.60 (0.54 to 0.67)	<0.001***	0.64 (0.58 to 0.71)	<0.001***
Age range						
Younger age (≤65 years)	838 (30.4)	1249 (30.8)	1.00 (REF)			
Recently retired (66–70 years)	1087 (39.4)	1742 (42.9)	1.08 (0.96 to 1.21)	0.225	1.05 (0.93 to 1.18)	0.47
Older population (≥71 years)	831 (30.2)	1070 (26.3)	0.86 (0.76 to 0.98)	0.023*	0.73 (0.64 to 0.80)	<0.001***
Older compared with recently retired	831 (30.2)	1070 (26.3)	0.80 (0.71 to 0.90)	<0.001***	0.70 (0.62 to 0.79)	<0.001***
Smoking status						
Current smoker	1334 (48.4)	1568 (38.6)	0.67 (0.61 to 0.74)	<0.001***	0.70 (0.63 to 0.78)	<0.001***
Ex-smoker	1418 (51.5)	2591 (61.3)	1.00 (REF)			
Never-smoked‡	4 (0.1)	2 (0.0)	(–)			
Socioeconomic group						
Quintile 1 (most deprived)	924 (33.5)	1090 (26.8)	0.52 (0.45 to 0.60)	<0.001***	0.56 (0.49 to 0.65)	<0.001***
Quintile 2	448 (16.3)	487 (12.0)	0.48 (0.40 to 0.57)	<0.001***	0.49 (0.42 to 0.59)	<0.001***
Quintile 3	483 (17.5)	723 (17.8)	0.66 (0.56 to 0.77)	<0.001***	0.68 (0.58 to 0.80)	<0.001***
Quintile 4	447 (16.2)	732 (18.0)	0.72 (0.61 to 0.85)	<0.001***	0.73 (0.62 to 0.86)	<0.001***
Quintile 5 (least deprived)	453 (16.4)	1029 (25.3)	1.00 (REF)			

*p<0.05, ***p<0.001.

†Adjusted for all other variables in model.

‡Smoking status data <2% excluded from statistical analyses.

Table 2 compares affective risk perception between non-uptake NPQ completers and positive uptake groups. Individuals with a higher affective risk perception were less likely to take part in lung cancer screening, when compared with individuals reporting no or lower levels of affective risk perception (OR=0.52, p<0.001 and OR=0.64, p<0.001, respectively, after adjusting for age, gender, smoking status and socioeconomic group). There was no statistically significant difference between none and lower levels of affective risk perception.

**Table 2:** Univariable and multivariable analyses of level of affective risk perception influencing lung screening uptake in high-risk individuals

	NPQ completers (n=748) n (%)	Positive uptake (n=4061) n (%)	Univariable OR (95% CI)	p Value	Multivariable OR (95% CI) †	p Value
Affective risk perception						
None (not at all concerned)	129 (17.2)	1054 (26.0)	1.00 (REF)			
Lower (somewhat concerned)	213 (28.5)	1493 (36.8)	0.86 (0.69 to 1.09)	0.219	0.82 (0.65 to 1.04)	0.094
Higher (moderately or very concerned)	329 (44.0)	1478 (36.4)	0.55 (0.45 to 0.69)	<0.001***	0.52 (0.42 to 0.65)	<0.001***
Higher compared with lower	329 (44.0)	1478 (36.4)	0.64 (0.53 to 0.77)	<0.001***	0.64 (0.53 to 0.77)	<0.001***
Missing	77 (10.3)	36 (0.9)	(–)			

***p<0.001.

†Adjusted for age, gender, smoking status and socioeconomic group.

NPQ, non-participation questionnaire.

### Effects of NPQ completion versus non-completion

Among high-risk individuals declining participation in the UKLS trial, older individuals were more likely than younger individuals to complete the NPQ (OR=2.15, p<0.001), as were ex-smokers compared with current smokers (OR=1.49, p<0.001) (see online supplementary appendix S2). Individuals of the lowest socioeconomic group were less likely to complete the NPQ compared with individuals of the highest socioeconomic group (OR=0.65, p=0.001).

### Self-reported barriers to participation among high-risk non-uptake individuals

Six overarching themes were identified: practical barriers, emotional barriers, age, trial acceptability, low perceived risk and dislikes. An overview of the different themes and subcategories is shown in table 3, with illustrative quotes provided for the two main themes reflecting practical barriers and emotional barriers. The κ coefficient was 0.88 and discrepant coding was resolved through discussion.

**Table 3:** Self-reported reasons for non-participation in the UKLS trial

Theme	NPQ completers (n=748) n (%)	Subcategory*	n
Practical barriers	350 (46.8)	Travel	138
		Comorbidities	120
		Carer responsibilities	43
		Already receiving scans	41
		Work and other commitments	23
		Not in area	20
		Taking part in other research	8
		Language or literacy problems	6
		Cannot be scanned	4
		Prior exposure to radiation	3
		Effort required	5
Emotional barriers	138 (18.4)	Avoidance of lung cancer information	17
		Fear	15
		Anxiety from taking part or results	6
		Mistrust of medical system	2
		Recent bereavement	2
		Anxiety of family member	1
Trial acceptability	18 (2.4)	Trial acceptability	1
		Duration or frequency	11
		Unwilling to be randomised	6
Age	16 (2.1)	Age	10
		Too old	6
Dislikes	13 (1.7)	Hospital or healthcare system	9
		Scans and tests	4
Low perceived risk	12 (1.6)	Low perceived risk	12
Other	30 (4.0)	No reason stated	23
		Already have/had lung cancer	4
		Would like to take part	2
		Thought request was for partner	1

*Some individuals provided more than one answer, and were therefore counted in more than one subcategory.

NPQ, non-participation questionnaire; UKLS, UK Lung Cancer Screening.

#### Practical barriers

The most commonly reported reasons for non-participation in high-risk non-uptake individuals were practical barriers (see box 1), including *travel* with difficulties relating to the distance of travel, lack of public transport available, and the cost of either the journey itself or hospital parking. *Comorbidities* were also a commonly reported practical barrier to participation, with individuals feeling that either their current comorbidity or related treatments prevented them from attending hospital to participate in the trial. Other commonly reported practical barriers included *carer responsibilities, already receiving screening,* and *not being in the area*.

Box 1 **Practical barriers**

“*I would like to take part but I do not have a car and it is very difficult to get to Papworth—involves train and taxi or two buses and a couple of hours each way”*

“I am being admitted to hospital on December 2nd 2011 for hip replacement otherwise I would have been happy to participate”

“*I have a heart problem and gastric problems and in the last 3 years I have had CT scans and X-rays, and I am going to have another CT scan in Feb this year 2012. So it’s for these reasons that I do not wish to take part”*

#### Emotional barriers

Emotional barriers most commonly included *avoidance of lung cancer information* and *fear* (see box 2).

Box 2 **Emotional barriers**

“*I do not wish to know if I had lung cancer, so I try not to think about it”*

“Frightened”

“*Would be anxious and worried about actually taking part in the physical research project. Sorry”*

#### Trial acceptability, age, dislikes and low perceived risk

*Trial acceptability* was mentioned as a reason for non-participation—some individuals felt that the duration or frequency of the trial was not practical, and others did not want to take part as there was potential to not receive the intervention of a LDCT scan. *Age* was also described as a reason for not taking part, with some individuals stating that they were above the desired age range (50–75 years), while others felt that they were “*too old*”. Some individuals mentioned *dislikes* for hospitals, healthcare system, or scans and tests. *Low perceived risk* was also reported, with most of these responses relating to either no longer smoking or smoking too few cigarettes to warrant lung cancer screening.

### Exploratory associations between risk factors and self-reported barriers to participation

Among those declining to participate, the odds of reporting travel as a barrier were more than double in quintiles 3–5 when compared with quintile 1 (OR=2.37, p=0.005; OR=2.91, p<0.001; OR=2.25, p=0.009, respectively) (see online supplementary appendix S3). Individuals with a higher affective risk perception were more likely to report comorbidities as a barrier to participation (OR=1.84, p=0.005). Smokers were less likely to report practical barriers such as already receiving scans, work/other commitments and not being in the area (OR=0.48, p=0.002), and more likely to report emotional barriers as reasons for non-participation (OR=2.02, p=0.013) compared with ex-smokers. Emotional barriers were also more likely to be reported by older individuals (OR=2.94, p=0.036). Associations between gender and self-reported barriers were not statistically significant.

## Discussion

To the best of our knowledge, the current study is the first to use a mixed methods approach to examine the barriers to participation among high-risk individuals in a lung cancer screening trial. A profile of potential risk factors for non-uptake of lung screening was revealed. High-risk individuals who were older, female, smokers, from a lower socioeconomic group, or with a higher affective risk perception were less willing to participate in the UKLS trial. Practical barriers reflecting difficulties with travelling to attend screening, comorbid illnesses and treatments, and carer responsibilities were the most common self-reported reasons for non-participation. Exploratory analysis revealed that travel was a more commonly reported barrier among individuals of higher socioeconomic group, and individuals with a higher affective risk perception more commonly reported barriers relating to comorbidities. Smokers were more likely to report emotional barriers as reasons for non-participation.

At the initial stage of recruitment from the general population into the UKLS trial, uptake generally increased with age, with the exception of individuals aged ≥71 years where uptake was the lowest.^7^ In contrast, no difference in screening uptake was observed between younger and recently retired high-risk individuals in the current study, although older individuals were less likely to participate in screening. Reduced uptake among older individuals in both stages of the UKLS trial is important, since more than half of lung cancer cases occur in individuals aged over 70 years.^23^

High-risk women were less likely than high-risk men to participate in the UKLS trial. Currently the only UK screening programme that recruits both genders is the colorectal screening programme. Although initial uptake of faecal occult blood testing is higher among women, they are less likely than men to take further testing involving sigmoidoscopy.^24–26^ It can be argued that being recalled for flexible sigmoidoscopy infers high risk similar to that of being invited back to participate in the UKLS trial; both of these may provoke fear and concerns related to lung cancer. Previous studies have found cancer-related fears and concerns to be more prevalent among women than men.^27^ ^28^

The association between smoking status and trial uptake is consistent with previous studies that revealed lower uptake in lung cancer screening among smokers.^7^ ^11^ ^12^ Exploratory analyses found that smokers were more likely to report emotional than practical barriers when compared with ex-smokers, reflecting fear, anxiety and a wish to avoid lung cancer-related information. Similarly, Silvestri *et al*^11^ found that when compared with never-smokers, current smokers reported more fatalistic attitudes and were less likely to believe that early detection and intervention would result in a good chance of survival.

In contrast to van den Bergh *et al*^10^ who found that a higher affective risk perception was a motivator for lung screening in the NELSON, the current study found that high-risk individuals with a higher affective risk perception were less likely to participate in the UKLS trial. It is likely, therefore, that a high-risk status was inferred, which for some individuals induced avoidance of cancer-related information. This is consistent with previous studies which report that higher levels of concern or threat associated with personal cancer risk may act as a deterrent to screening through the mechanism of avoidance.^16^ ^17^

The association between lower socioeconomic group and lower screening uptake among high-risk individuals echoes that of previous studies.^8^ ^9^ ^29^ The effect of socioeconomic deprivation on screening uptake has been attributed in part to fearful and fatalistic beliefs among more deprived populations.^30^ ^31^ Such beliefs may partly stem from, and be reinforced by, greater exposure to lung cancer and other prevalent respiratory diseases in deprived communities as a consequence of socioeconomic differences in tobacco use.^32^ ^33^

Previous studies have found travel to be an important barrier to participation in cancer screening.^12^ ^34^ In the current study, difficulties with travel were more commonly reported by higher, rather than lower, socioeconomic groups. However, we suggest that this counterintuitive finding is a confound of geographical area rather than a true effect, reflecting difficulty in travelling to Papworth Hospital—located in rural and more affluent Cambridgeshire—compared with Liverpool Heart and Chest Hospital.^35^

The use of a mixed methods approach allowed for a richer exploration of the barriers to lung screening uptake than quantitative or qualitative analysis alone.^36^ In addition, the use of free-text comments in a questionnaire provided an opportunity for data to be gathered from a large sample of individuals, and from those who may not be willing or able to participate in interviews or focus groups.^37^ ^38^ However, methodological issues associated with response bias are acknowledged. Younger individuals, those from lower socioeconomic groups, and smokers were less likely to complete the NPQ. As a result, there was an under-representation of self-reported barriers in these individuals. In addition, individuals may not be consciously aware of underlying motivations for their behaviour.^39^ Some barriers might be perceived as more legitimate than others, and emotional barriers relating to avoidance, fear and anxiety may, therefore, have been under-reported. Further qualitative studies are needed to gain an in-depth understanding of barriers and facilitators to lung cancer screening in high-risk individuals.

Smoking status was computed from self-reported information; so there is potential for smokers to be under-represented, although previous studies have shown the validity of self-reported smoking status to be high.^40^ ^41^ Affective risk perception was assessed using one item from the revised Cancer Worry Scale.^20^ ^21^ The use of this single-item measure in predicting level of affective risk perception has not yet been validated.

Although randomised trials are the gold standard for evidence-based decision-making in medicine,^42^ an individual's decision about participating in a trial is different from deciding to participate in a national screening programme. As a result, there may be limitations regarding the generalisability of results to the general population when a national lung cancer screening programme is implemented in practice, and also a risk of amplifying the effects of sociodemographic variables on non-uptake, as observed in the current trial.

## Conclusion

Strategies to improve equitable uptake are critical to the successful implementation of new cancer screening programmes. The current study highlighted important subgroups who were less likely to take part in UKLS trial and in whom lung cancer risk is known to be higher.^23^ In the case of a national lung cancer screening programme, efforts to improve uptake should include strategies for engaging women and those most at risk, including adults over 70 years, smokers, and those from deprived areas. Practical barriers relating to access should be addressed, with behavioural interventions designed to minimise emotional barriers, especially among current smokers.
